# Supplementary material for: Serum Metabolomics Profiling Reveals Metabolic Alterations Prior to a Diagnosis with Non-Small Cell Lung Cancer among Chinese Community Residents: A Prospective Nested Case-Control Study
Source: Metabolites. 2022 Sep 27;12(10):906. doi: 10.3390/metabo12100906 (PMC9610639; doi:10.3390/metabo12100906)
Supplement: Supplementary file 1 [file metabolites-12-00906-s001.zip › metabolites-1896884-supplementary.pdf]

Supplementary Materials

Table S1: NSCLCs vs Cancer-free controls: Differentially expressed metabolites (ONLY named presented)

ID	Compound Name	MS2 Score	rt	mz	Platform	VIP	P-value	Q-value	FC	LogFC	Expression
1	N1-Methyl-4-pyridone-3-carboxamide	0.99	47.34	153.07	LC/MS-POS	2.96	0.00	0.00	1.37	0.14	upward
2	Tigloidine	0.98	686.46	224.16	LC/MS-POS	1.97	0.01	0.01	0.77	-0.11	downward
3	L-Palmitoylcarnitine	0.94	199.30	400.34	LC/MS-POS	1.76	0.02	0.05	1.17	0.07	upward
4	N,N'-Diacetylhydrazine	0.91	255.24	117.07	LC/MS-POS	1.91	0.01	0.06	0.91	-0.04	downward
5	(3xi,6xi)-Cyclo(alanylvalyl)	0.90	283.28	171.11	LC/MS-POS	3.59	0.00	0.00	0.70	-0.15	downward
6	D-1-Amino-2-pyrrolidinecarboxylic acid	0.87	326.82	131.08	LC/MS-POS	2.77	0.00	0.00	0.79	-0.10	downward
7	Artonin C	0.83	59.79	679.26	LC/MS-POS	2.68	0.00	0.01	2.91	0.46	upward
8	Hexacosanoyl carnitine	0.81	186.46	540.50	LC/MS-POS	1.57	0.01	0.01	1.31	0.12	upward
9	PC(22:6/20:3)	0.80	153.92	856.58	LC/MS-POS	1.77	0.02	0.03	0.87	-0.06	downward
10	2,6-Dimethoxy-4-(1-propenyl)phenol	0.76	405.79	195.10	LC/MS-POS	3.82	0.00	0.00	0.81	-0.09	downward
11	PC(22:6/20:2)	0.73	153.99	858.60	LC/MS-POS	2.32	0.00	0.01	0.83	-0.08	downward
12	LysoPC(18:2(9Z,12Z))	0.70	216.20	520.34	LC/MS-POS	2.63	0.00	0.00	0.85	-0.07	downward
13	Sphingosine	0.69	82.04	300.29	LC/MS-POS	1.49	0.02	0.05	1.41	0.15	upward
14	PC(20:4/20:3)	0.68	155.73	832.59	LC/MS-POS	2.16	0.00	0.06	0.83	-0.08	downward
15	Na,Na-Dimethylhistamine	0.67	378.38	140.12	LC/MS-POS	3.21	0.00	0.00	0.66	-0.18	downward
16	Glucosylceramide (d18:1/24:1(15Z))	0.57	40.24	810.68	LC/MS-POS	2.08	0.01	0.02	0.80	-0.10	downward
17	PC(22:6/20:4)	0.53	151.41	854.57	LC/MS-POS	1.90	0.02	0.04	0.84	-0.08	downward
18	Pipericine	0.47	34.05	336.33	LC/MS-POS	1.13	0.01	0.05	0.79	-0.10	downward

Table S2. NSCLCs vs Cancer-free controls: KEGG Pathway Enrichment Analysis

ID	KEGG ID	Pathway	Matched	Total	P-value	LogP	Background Ratio	Metabolite Ratio	Fold Enrichment
1	hsa05231	Choline metabolism in cancer	2	11	0.00	3.07	0.00	0.14	44.18
2	hsa00600	Sphingolipid metabolism	2	21	0.00	2.50	0.01	0.14	23.14
3	hsa00564	Glycerophospholipid metabolism	2	36	0.01	2.04	0.01	0.14	13.50
4	hsa04210	Apoptosis	1	4	0.02	1.79	0.00	0.07	60.75
5	hsa00591	Linoleic acid metabolism	1	5	0.02	1.69	0.00	0.07	48.60
6	hsa04217	Necroptosis	1	10	0.04	1.39	0.00	0.07	24.30
7	hsa04071	Sphingolipid signaling pathway	1	15	0.06	1.22	0.00	0.07	16.20
8	hsa00760	Nicotinate and nicotinamide metabolism	1	15	0.06	1.22	0.00	0.07	16.20
9	hsa04723	Retrograde endocannabinoid signaling	1	19	0.08	1.12	0.01	0.07	12.79
10	hsa00590	Arachidonic acid metabolism	1	36	0.14	0.86	0.01	0.07	6.75
11	hsa01212	Fatty acid metabolism	1	39	0.15	0.83	0.01	0.07	6.23
12	hsa00983	Drug metabolism - other enzymes	1	39	0.15	0.83	0.01	0.07	6.23
13	hsa00592	alpha-Linolenic acid metabolism	1	42	0.16	0.80	0.01	0.07	5.79
14	hsa00071	Fatty acid degradation	1	50	0.19	0.73	0.01	0.07	4.86

Metabolite ratio = the number of metabolite enriched in the pathway / the total number of significantly changed metabolites matched with HMDB database and involved in the KEGG pathway;.
Background ratio = the number of metabolite enriched in the pathway / the total number of metabolites involved in human metabolic reaction;.
Fold enrichment ratio = metabolite ratio / background ratio;.
